# Identification and evolutionary dynamics of two novel human coronavirus OC43 genotypes associated with acute respiratory infections: phylogenetic, spatiotemporal and transmission network analyses

**DOI:** 10.1038/emi.2016.132

**Published:** 2017-01-04

**Authors:** Xiang Yong Oong, Kim Tien Ng, Yutaka Takebe, Liang Jie Ng, Kok Gan Chan, Jack Bee Chook, Adeeba Kamarulzaman, Kok Keng Tee

**Affiliations:** 1 Department of Medical Microbiology, Faculty of Medicine, University of Malaya, 50603 Kuala Lumpur, Malaysia; 2 Department of Medicine, Faculty of Medicine, University of Malaya, 50603 Kuala Lumpur, Malaysia; 3 AIDS Research Center, National Institute of Infectious Diseases, 162-8640 Tokyo, Japan; 4 Faculty of Information Science & Technology, Multimedia University, 75450 Melaka, Malaysia; 5 Division of Genetics and Molecular Biology, Institute of Biological Sciences, Faculty of Science, University of Malaya, 50603 Kuala Lumpur, Malaysia

**Keywords:** comparative genomic analyses, evolutionary dynamics, human coronavirus OC43, recombination, transmission network

## Abstract

Human coronavirus OC43 (HCoV-OC43) is commonly associated with respiratory tract infections in humans, with five genetically distinct genotypes (A to E) described so far. In this study, we obtained the full-length genomes of HCoV-OC43 strains from two previously unrecognized lineages identified among patients presenting with severe upper respiratory tract symptoms in a cross-sectional molecular surveillance study in Kuala Lumpur, Malaysia, between 2012 and 2013. Phylogenetic, recombination and comparative genomic analyses revealed two distinct clusters diverging from a genotype D-like common ancestor through recombination with a putative genotype A-like lineage in the non-structural protein (nsp) 10 gene. Signature amino acid substitutions and a glycine residue insertion at the N-terminal domain of the S1 subunit of the spike gene, among others, exhibited further distinction in a recombination pattern, to which these clusters were classified as genotypes F and G. The phylogeographic mapping of the global spike gene indicated that the genetically similar HCoV-OC43 genotypes F and G strains were potentially circulating in China, Japan, Thailand and Europe as early as the late 2000s. The transmission network construction based on the TN93 pairwise genetic distance revealed the emergence and persistence of multiple sub-epidemic clusters of the highly prevalent genotype D and its descendant genotypes F and G, which contributed to the spread of HCoV-OC43 in the region. Finally, a more consistent nomenclature system for non-recombinant and recombinant HCoV-OC43 lineages is proposed, taking into account genetic recombination as an important feature in HCoV evolution and classification.

## Introduction

Human coronavirus OC43 (HCoV-OC43), belonging to the *Betacoronavirus* genus of the Coronaviridae family,^1^ continues to cause respiratory tract infections in children and adult populations worldwide.^2, 3^ HCoV-OC43 and other human coronaviruses (HKU1, NL63, 229E, SARS-CoV and MERs-COV) contain a large positive-sense single-stranded RNA with a genome size from ~27 to 31 kb.^4^ Previous studies have focused on investigating the molecular epidemiology of HCoV-OC43 to understand its evolution and pathogenicity.^5, 6, 7, 8, 9, 10, 11^ HCoVs continue to evolve through homologous RNA recombination and exhibit high nucleotide substitution rates across the genome,^12, 13^ resulting in the emergence of novel variants that can adapt to new hosts or ecological niches.^14, 15, 16, 17, 18^

Since the first description of HCoV-OC43 in the 1960s, five genetically distinct genotypes (A through E) have been identified based on phylogenetic analysis of main genes, such as the spike (S), RNA-dependent RNA polymerase (RdRP) and nucleocapsid (N) genes and complete viral genome.^7, 9^ Genotypes A and B were estimated to have emerged around the 1950s and 1990s, respectively, whereas genotypes C, D and E were detected more recently in the 2000s.^7, 9^ Genotype D arose from recombination between genotypes B and C and was dominant in parts of Asia and Europe.^7, 8, 9, 19^ Likewise, genotype E was generated from recombination among genotypes B, C and D in Asia,^9^ underlining the importance of recombination in driving the evolution of HCoV-OC43.

A cross-sectional molecular surveillance of HCoV-OC43 and HCoV-HKU1 was conducted among patients presented with acute upper respiratory tract infection (URTI) in Kuala Lumpur, Malaysia.^20^ Both HCoV-OC43 and HCoV-HKU1 were co-circulating throughout the year, but the lowest detection rates were reported between October and January,^20^ a period that coincides with the Northeast Monsoon season (November to March), which brings in more rainfall compared with the Southwest Monsoon.^21^ Interestingly, phylogenetic analysis of the partial S gene (S1 domain) revealed that a majority of the HCoV-OC43 strains shared a genotype D-like common ancestor but diverged into two unique clusters. In this study, we obtained the full-length genome sequences of these unique strains and performed phylogenetic and recombination analyses, suggesting a possible emergence of two novel recombinant genotypes descended from genotype D, which were designated as genotypes F and G. Through a database search of global S gene sequences, Bayesian coalescent phylogenetic and amino acid sequence analyses implied that these two novel genotypes were likely to have emerged around the late 2000s to early 2010s with a wide geographical dispersion. Their origins were probably mapped to Asia where the putative parent genotype D was circulating at high prevalence, driven in part by the emergence and persistence of multiple sub-epidemic transmission networks of respiratory tract infections.

## Materials and Methods

### Clinical specimens

This study was approved by the University of Malaya Medical Centre (UMMC) Medical Ethics Committee (MEC890.1). Standard, multilingual consent forms from the Medical Ethics Committee were used, and written consent was obtained from all study participants. A total of 2060 consenting outpatients presented with symptoms of acute URTI were recruited at the primary care clinics of University Malaya Medical Centre in Kuala Lumpur, Malaysia between March 2012 and February 2013. The nasopharyngeal swabs collected from the patients were transferred to the laboratory in universal transport media (Copan Diagnostics, Inc., Murrieta, CA, USA) and stored at −80 °C. The xTAG Respiratory Virus Panel (RVP) FAST multiplex RT-PCR assay (Luminex Molecular, Toronto, ON, Canada) and Luminex's proprietary Universal Tag sorting system on Luminex 200 IS platform (Luminex, Austin, TX, USA) were used to detect HCoV-OC43 in the samples according to the manufacturer's protocol.^22^ As reported previously, through phylogenetic analysis of the partial S gene (S1 domain), 21 out of 2060 nasopharyngeal samples (1.02%), which were positive for HCoV-OC43, formed two distinct clades provisionally designated as lineages 1 and 2 that shared a genotype D-like common ancestor.^20^

### Full-length genome sequencing

To characterize and evaluate the novelty of the two distinct HCoV-OC43 lineages, 16/21 strains (nine from lineage 1 and seven from lineage 2) from 16 infected patients were prepared for further whole-genome analysis. The demographic and clinical profile of patients infected with HCoV-OC43 lineages 1 and 2 are summarized in Table 1. To obtain the full-length genome of these unique strains, viral RNA was extracted by the NucliSENS easyMAG automated nucleic acid extraction system (bioMérieux, Marcy I'Etoile, France)^23^ and reversely transcribed into cDNA using SensiFAST cDNA Synthesis Kit (Bioline, London, UK), which contains anchored oligo(dT) and random hexamer primers. The full-length genome cDNA of ~30 kb in size (which flanks from the 5′ end of *ORF1a* gene to 3′ end of the poly-A tail) was amplified by a genome walking method that involved a total of 44 overlapping fragments using a set of previously published primers with minor modifications for improved sequence coverage (Supplementary Table S1).^9^ PCR thermocycling conditions were set as follows: initial denaturation at 95 °C for 1 min, 35 cycles of amplification at 95 °C for 15 s, 50 °C for 15 s and 72 °C for 30 s using the MyTaq HS Red Mix (Bioline, London, UK) kit. PCR products were purified, and sequencing reactions were performed in ABI PRISM 3730XL Genetic Analyzer using the BigDye Terminator v3.1 cycle sequencing kit chemistry (Applied Biosystems, Foster City, CA, USA). Finally, sequence reads were assembled into a contig and manually edited using BioEdit 7.2 (Ibis Therapeutics, Carlsbad, CA, USA) to produce a final sequence of full-length HCoV-OC43 genomes. All sequences generated in this study are available from GenBank under accession numbers KX538964–KX538979.

### Phylogenetic, recombination and amino acid sequence analyses

To determine the evolutionary relationship among the unique and global HCoV-OC43 strains, phylogenetic analysis was conducted using full-length genome sequences. All 16 unique sequences were first aligned with published global reference sequences (genotypes A to E) retrieved from GenBank (accessed on 31 March, 2016) (Supplementary Table S2) using a web-based multiple sequence alignment program MAFFT.^24^ Phylogenetic tree reconstruction using the neighbor-joining (NJ) method and inter-genotype pairwise genetic distance calculation for sequence divergence comparison were performed using MEGA 6.0.^25^ The maximum-likelihood (ML) method was also performed for reconstruction of a phylogenetic tree, which was heuristically inferred using subtree pruning and regrafting and nearest neighbor interchange algorithms with a general time-reversible (GTR) nucleotide substitution model, a proportion of invariant sites (+I) and four categories of gamma rate heterogeneity (+Γ_4_), which were implemented in PAUP version 4.0.^26^ Kimura's two-parameter model with a reliability of branching order analyzed by bootstrap replicates of 1000 was used. Subsequently, bootscanning was performed using SimPlot version 3.5.1 to determine possible recombination events and location of breakpoints in the viral genome of unique strains. This approach has been previously reported.^7, 9, 15, 27^ Sub-genomic regions located between recombination breakpoints were subjected to additional phylogenetic analysis using the neighbor-joining method to infer the recombination structure and the parental genotype of each region. Signature nucleotide and amino acid substitutions of the unique strains were determined by Sequence Data Explorer in MEGA.

### Estimation of divergence times

The Bayesian Evolutionary Analysis by Sampling Trees (BEAST) program has been widely used to investigate the spatiotemporal and evolutionary dynamics of viral pathogens using time-stamped nucleotide sequence data sets.^28^ Previously, estimations of divergence times of HCoV-OC43 strains relied mainly on the S gene sequence data^9, 10, 11^ given that the S protein is the major antigenic protein with high selection pressure and genetic diversity compared with other viral proteins.^4^ In this study, using the query (*n*=16) and global reference full-length genome sequences (*n*=13), the divergence times of all HCoV-OC43 genotypes and lineages 1 and 2^20^ were estimated to determine when these strains emerged. The divergence times were also re-estimated using all S gene sequences available in the public database (S1 domain: 23 644–25 125 nt). The estimation was performed by molecular clock dating analysis using the Bayesian Markov chain Monte Carlo (MCMC) coalescence method implemented in BEAST 1.7.^28^ Two parametric demographic models (constant and exponential population sizes) and one non-parametric model (Bayesian Skyline Plot (BSP)) coalescent tree priors were used to infer the viral phylogenies, nucleotide substitution rates and time of most recent common ancestor (tMRCA). The uncorrelated exponential relaxed, uncorrelated lognormal relaxed and strict molecular clock models were tested. Analyses were performed under the general time-reversible nucleotide substitution model with a proportion of invariant sites (GTR+I). MCMC runs for the full-length genome and S gene were 50 million steps long, with sampling every 50 000 states. Using Tracer version 1.6 (http://tree.bio.ed.ac.uk/software/tracer), the output was assessed for convergence by means of effective sampling size greater than 200 after a 10% burn-in. Bayesian maximum clade credibility (MCC) trees were annotated using the Tree Annotator program included in the BEAST package by choosing the tree with the maximum sum of posterior probabilities after a 10% burn-in. The final MCC trees were visualized in FigTree (http://tree.bio.ed.ac.uk/software/figtree/).

### Transmission network analysis of HCoV-OC43 genotype D and its related recombinants

As HCoV-OC43 genotype D has been the most prevalent and persistent genotype circulating in East Asia in recent years,^7, 9, 11^ an estimation of the transmission network of genotype D and its related recombinants^20^ could be a useful strategy to elucidate the degree of spread and dynamics of infection attributed to these genotypes within and between countries.^29, 30^ To deduce the transmission pattern of HCoV-OC43 genotype D and its related recombinants in recent years, a transmission cluster was deduced from new and published S gene sequences based on the Tamura-Nei 93 (TN93) pairwise distance estimates performed using a custom script in Python (release 3.2.6) with a bootstrap analysis of 1000 replicates.^29, 30^ In the present study, a transmission cluster is defined as a cluster consisting at least two individuals (nodes) whose viral sequences are genetically linked (edges) at a given genetic distance threshold supported by bootstrap value of >90%.^29^ The genetic distance threshold was determined between the highest and lowest values of the intra- and inter-patient patristic distances, respectively, measured in nucleotide substitutions per site.^31, 32^ Given that HCoV-OC43 causes acute respiratory tract infection and hinders the estimation of intra-patient viral genetic distance, the most probable threshold value was determined from the 95% confidence interval of the lower 0.025 percentile of the inter-patient genetic distances^29^ as calculated from globally available and published S gene reference sequences (*n*=27) (Supplementary Tables S3 and S4). HCoV-OC43 sequences from different patients with a patristic distance less than the estimated threshold were identified either as transmission dyads (consists of two nodes) or networks (more than two nodes),^33^ reflecting the transmission linkages and genetic relatedness of the infecting HCoV-OC43 strains.

## Results

### Phylogenetic analysis of unique HCoV-OC43 strains using full-length genome sequences

**Figure 1:**
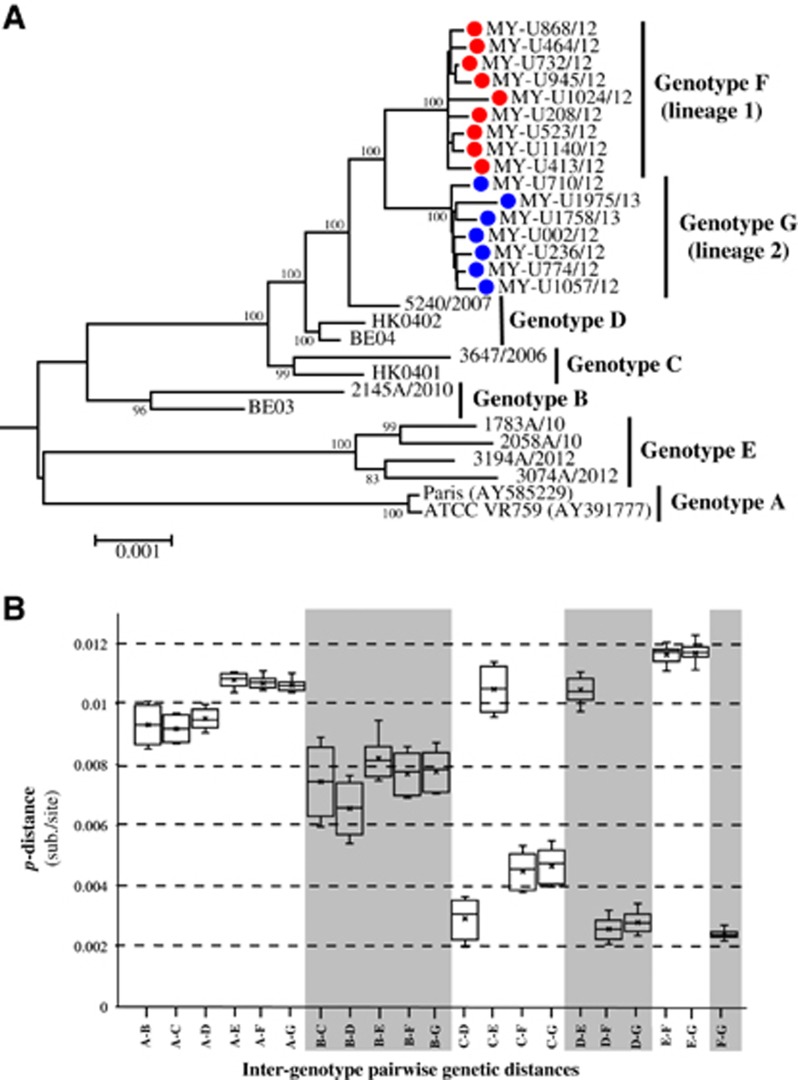
(**A**) Phylogenetic analysis of the HCoV-OC43 strains based on the full-length genome. Trees were reconstructed using the neighbor-joining method and Kimura 2-parameter model in MEGA 6.0. Bootstrap values were calculated from 1000 trees. Bootstrap values >70% were indicated on the branch nodes. The scale bar of an individual tree indicates the substitutions per site. (**B**) Estimation of pairwise genetic distances between HCoV-OC43 genotypes based on the full-length genome sequences. Genotypes F and G were previously classified as lineages 1 and 2, respectively.^20^

The phylogenetic tree reconstructed by the NJ method for the full-length genome is illustrated in Figure 1A, which consists of the unique Malaysian HCoV-OC43 strains and all available global reference sequences from 2001 to 2013 (except for prototype strain ATCC VR759, which was isolated in the 1960s) (Supplementary Table S2). These reference sequences were classified previously as genotypes A to E, and these reference viruses were isolated from patients with acute respiratory tract infection (ARTI) in Paris,^34^ Belgium,^5^ China^9^ and Hong Kong.^7^ Phylogenetic trees were also reconstructed by the NJ and ML methods for full-length genome, which include genotyped (published, *n*=13) and un-genotyped (unpublished, *n*=76) reference sequences as well as 2 cell-adapted/neurovirulent strain sequences, as shown in Supplementary Figure S1. Two distinct clusters (lineages 1 and 2) appeared to branch out from a genotype D-like common ancestor with high bootstrap support (100%) (Figure 1A; Supplementary Figure S1). The topology of this phylogenetic tree based on a full-length genome was similar to the tree topology based on a partial S gene reported in a previous study.^20^ On the basis of the estimation of inter-genotype pairwise genetic distances (Figure 1B), the distances of lineages 1 and 2 compared with genotypes A, B and E were >0.7% (0.007 substitutions/site), whereas distances were <0.5% when compared with genotypes C and D. This finding indicates that lineages 1 and 2 were more similar to genotypes C and D compared with genotypes A, B and E. Although the low mean genetic distance of 0.29±0.03% between genotypes C and D is probably attributed to the recombinant nature of genotype D (genotype D was generated from recombination between genotypes B and C),^7^ it is the lowest reported full-length genetic distance that separates HCoV-OC43 genotypes. Using this benchmark, with a genetic distance of 0.26±0.02% between genotype D and lineage 1 and 0.27±0.02% between genotype D and lineage 2, it is suggestive that the two lineages, which are designated as genotypes F and G hereafter, may have arisen and diverged from genotype D.

### Mosaic recombination structures of HCoV-OC43 genotypes F and G

**Figure 2:**
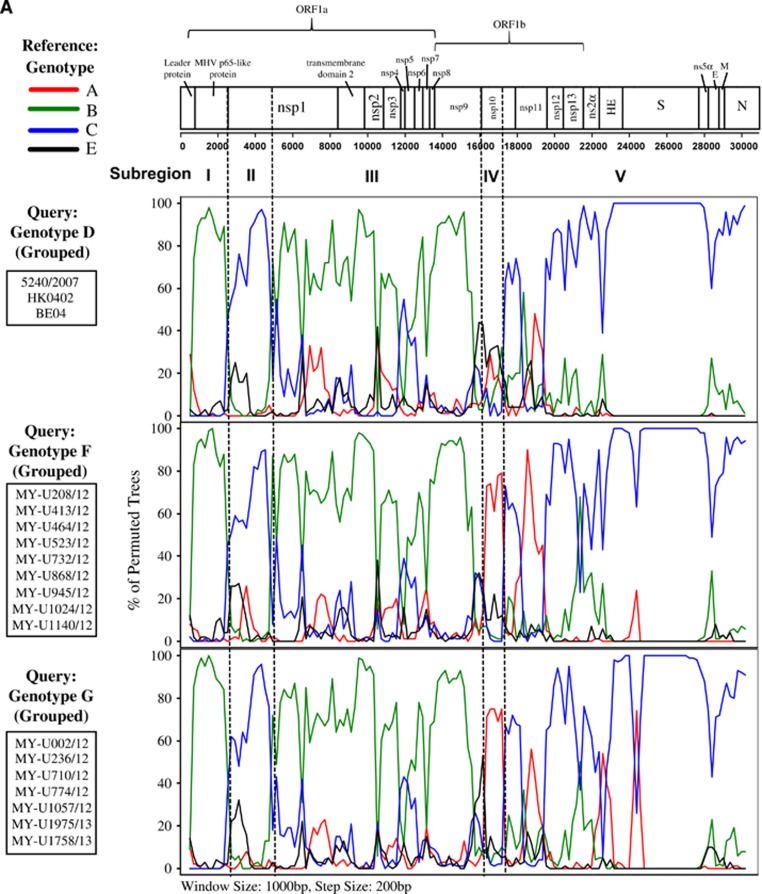

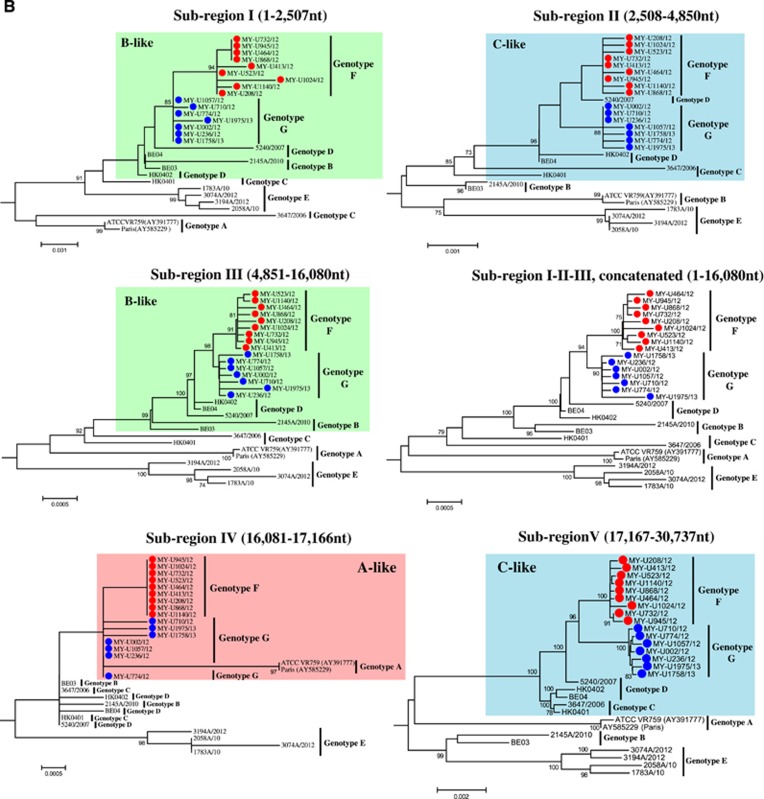
(**A**) Comparison of the mosaic recombination structure of the full-length genome between genotypes D, F and G. Bootscan analysis was performed using published and genotyped reference genomes for genotypes A, B, C and E as putative parental genotypes. (**B**) Putative parental genotype determination and confirmation in sub-genomic regions (sub-regions I–V) of genotypes D, F and F recombinants using the neighbor-joining method. Breakpoints determined by informative site analysis. Red, green and blue shades indicate genotypes A, B and C putative parental genotypes, respectively. Numbering of nucleotide (nt) positions is based on prototype ATCC VR759 reference strain.

The mosaic recombination structures of genotypes F and G were determined and compared with genotype D by performing bootscan analysis (sliding window size: 1000 bp, step size: 200 bp).^7, 9^ Published reference full-length genomes for genotypes A (prototype strain ATCC VR759, Paris strain—AY585229), B (Belgium 2003, 2145/2010), C (HK0401, 3647/2006) and E (1783A/10, 2058A/10, 3194A/12, 3074A/12) were used as putative parental genotypes. When the genomes of grouped genotypes F and G strains were used as query sequences, several potential recombination sites in the viral genomes were observed, separating the genome into at least five sub-regions (Figure 2A). From the 5′ end of the genome to position 16 080 nt, bootscan analysis showed that sub-regions I (positions 1 to 2507 nt) and III (4851 to 16 080 nt) of genotypes F and G were closely related to genotype B, whereas sub-region II (2508 to 4850 nt) was closely related to genotype C (supported by sub-region NJ trees in Figure 2B). Bootscan and sub-region tree analyses also revealed that these regions (sub-regions I–II–III) shared high homology with genotype D. From positions 16 081 to 17 166 nt (sub-region IV), genotypes F and G were closely related to genotype A, whereas positions 17 167 to 30 737 nt (sub-region V) were grouped with genotypes C and D (Figures 2A and 2B). When the mosaic recombination structures of genotypes F and G were compared with genotype D, it is noticeable that all three genotypes shared similar recombination breakpoints between 2500–3000 nt, 4500–5000 nt and 16 000–17 000 nt. However, both genotypes F and G had an additional recombination breakpoint between 17 000–17 500 nt, which was not observed in genotype D; thus, parts of the nsp10 gene (sub-region IV, 16 081–17 166 nt) were genotyped as A-like. This finding indicates that recombination events led to the emergence of novel genotypes F and G with a putative genotype A-like parental strain in the nsp10 region despite sharing similar recombination structure in most parts of the genome with genotype D strains.

### Nucleotide and amino acid sequence analysis

**Figure 3:**
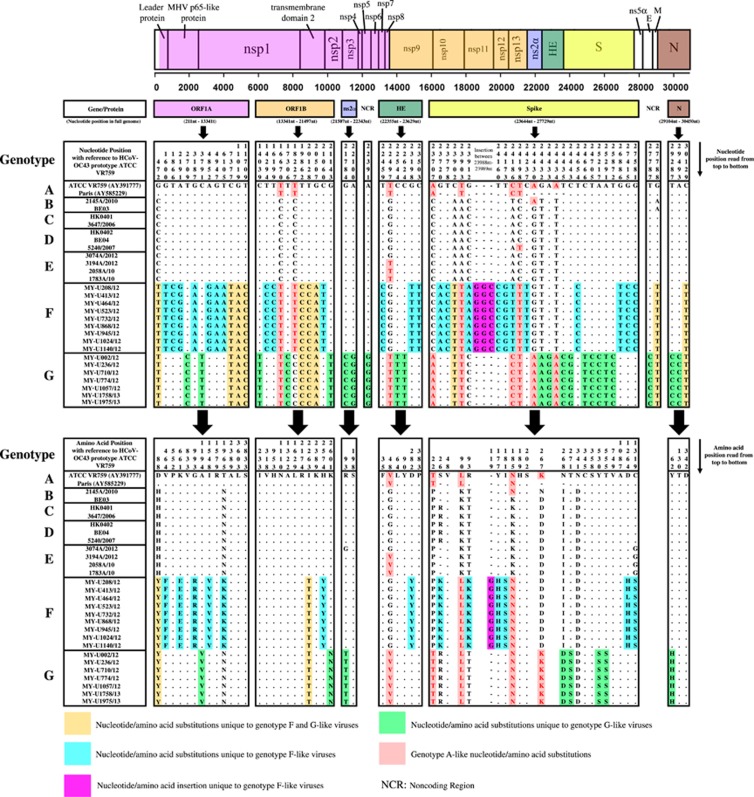
Signature nucleotide and amino acid substitution differences across the whole genome between genotypes F and G strains. Nucleotide and amino acid positions are numbered with a reference to HCoV-OC43 prototype strain ATCC VR759.

The whole genome of genotypes F and G strains was further subjected to nucleotide and amino acid sequence analysis to detect signature substitutions in their respective genomes given that the mosaic recombination pattern between genotypes F and G could not be clearly distinguished. However, both were evidently distinct from genotype D (Figure 2). As observed in Figure 3, using the prototype ATCC VR759 as the reference strain for nucleotide and amino acid positions, 34 and 32 nucleotide substitutions unique to genotypes F and G, respectively, were mapped across the whole genome. Corresponding to these nucleotide substitutions, 15 and 10 non-synonymous amino acid substitutions were observed in genotypes F and G, respectively. Of note, all genotype F strains had a unique 3-nucleotide insertion (GGC) between 23 988 and 23 989 nt that was not observed in genotype G as well as other genotypes, resulting in a glycine insertion at position 119 in the S protein. Likewise, genotype A-like nucleotide substitutions were observed in the S gene at positions 23 707, 24 186, 24 430 and 24 434 nt in genotype G (but not in genotype F) (Figure 3), indicating a plausible recombination event in genotype G that involved genotype A. Altogether, these findings indicated that genotypes F and G had their respective distinctive genotypic features at the nucleotide and amino acid levels. Collectively, with the phylogenetic clustering, pairwise genetic distance, recombination and comparative genomic analyses between genotypes indicate that both genotypes F and G represent two distinct HCoV-OC43 genetic lineages that have descended from the genotype D parental lineage through recombination with a genotype A-like lineage.

### Global circulation and divergence times of genotypes F and G

**Figure 4:**
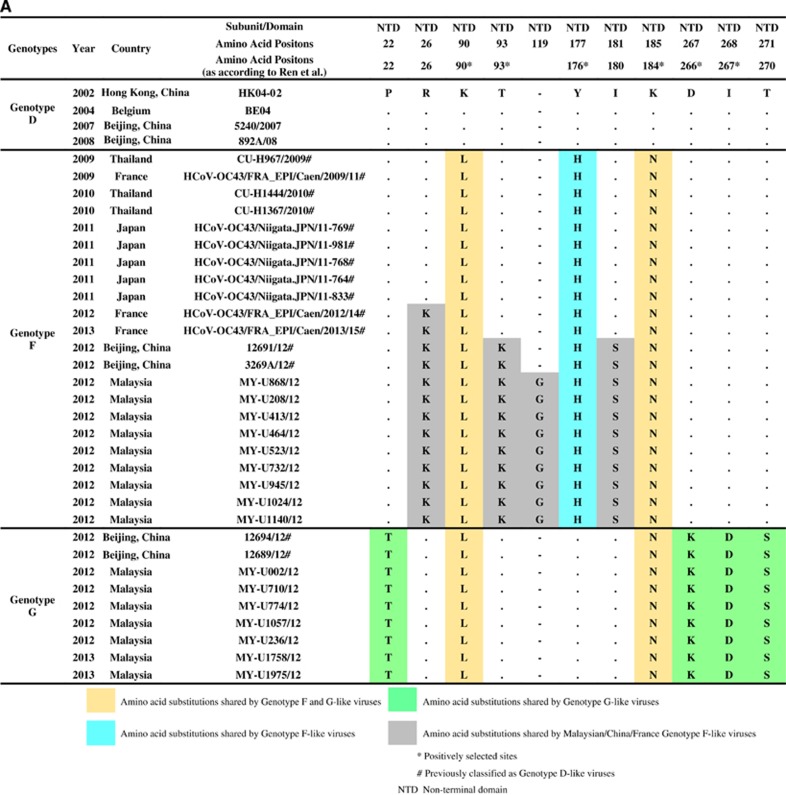

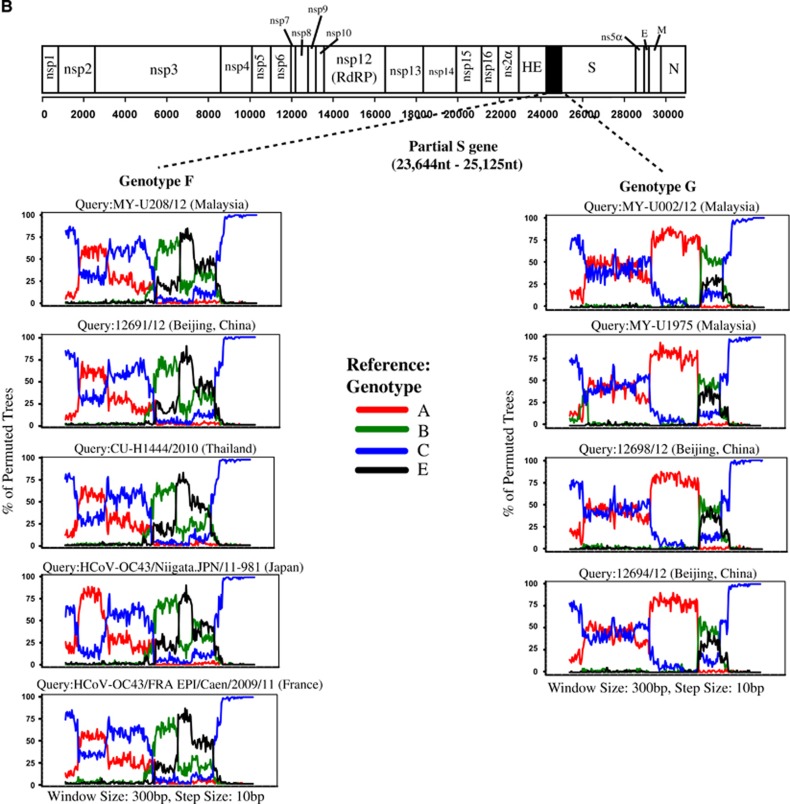
Evidence from (**A**) amino acid substitutions and (**B**) bootscan analysis on the partial S gene data to confirm the presence of genotypes F and G strains. The amino acid sequence and bootscan analyses are performed on the partial S gene region (23 644–25 125 nt). Bootscan analysis is performed with a window and step size of 300 and 10 bp, respectively.

**Figure 5:**
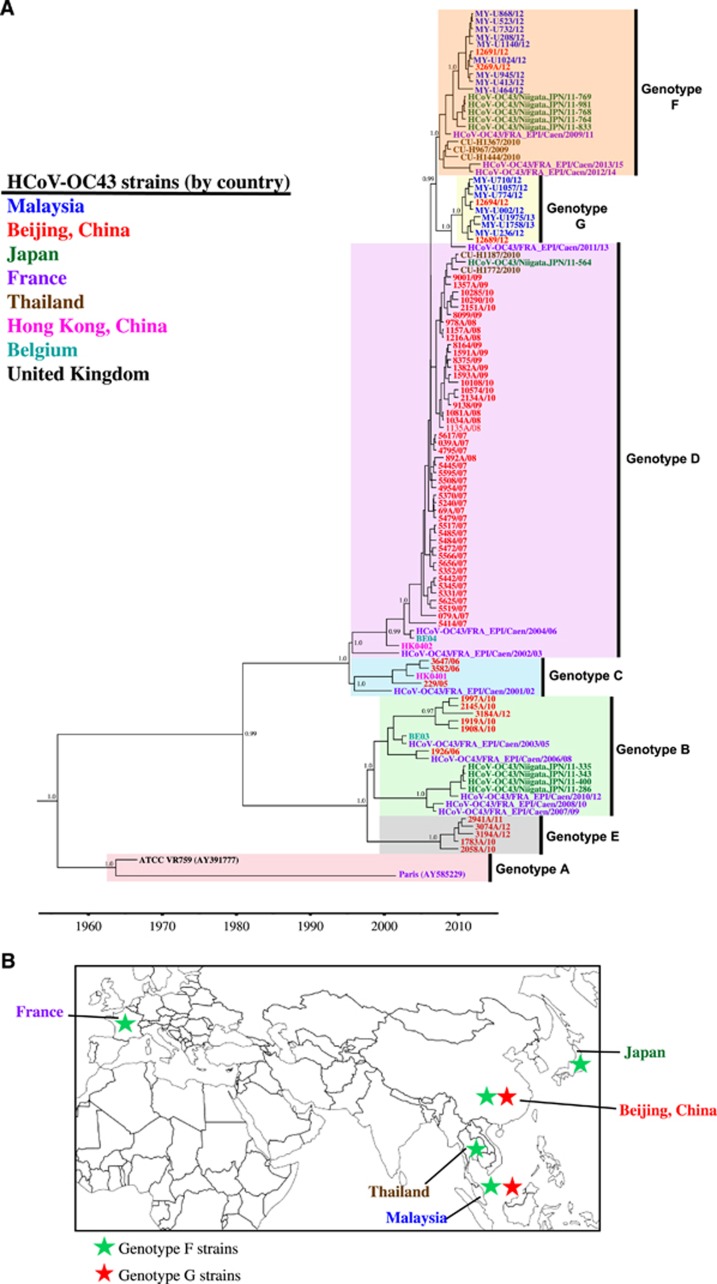
(**A**) Maximum clade credibility (MCC) tree of HCoV-OC43 strains based on the 114 complete and partial global S gene data. MCC posterior probability values were indicated on the nodes of each genotype. (**B**) Global distribution of genotypes F and G strains.

Phylogenetic analysis of the partial S gene (S1 domain) in a previous study demonstrated that several HCoV-OC43 strains from China (*n*=2), Thailand (*n*=3) and Japan (*n*=5) were clustered together with genotype F (previously known as lineage 1), whereas another two strains from China were clustered with genotype G (lineage 2).^20^ When amino acid sequence analysis was performed on the global partial S gene (S1 domain: 23 644 to 25 125 nt) in this study (using HCoV-OC43 genotype D as the reference strain), these genotype F strains from China, Thailand, Japan, and three newly deposited sequences from France (clustered together with genotype F strains in the MCC tree in Figure 5A) shared a signature amino acid substitution Y176H with the newly sequenced genotype F strains from Malaysia (Figure 4A). In addition, amino acid substitutions R26K, T93K and I181S, which were observed in genotype F strains from Malaysia, were also present in strains reported in China and/or France. In addition, it is interesting to note that the glycine insertion at position 119 was only present in the Malaysian strains. Though, despite this unique insertion, the bootscan analysis of the partial S gene (using a narrow sliding window of 300 bp and step size of 10 bp for improved resolution) revealed that all genotype F strains in these countries shared similar mosaic recombination structure (Figure 4B). The same could be observed for genotype G strains from China (*n*=2), which shared four signature amino acid substitutions P22T, D267K, I268D and T271S and similar mosaic recombination pattern in the partial S gene with the Malaysian strains (Figures 4A and 4B). In general, the amino acid and bootscan analyses of the partial S gene indicated that genetically similar HCoV-OC43 genotypes F and G strains can be found circulating in a number of Asian countries and Europe (Figure 5B).

To investigate the spatiotemporal and evolutionary dynamics of all HCoV-OC43 genotypes, divergence times were estimated by performing molecular clock dating analysis on 29 full-length genome sequences (including 16 new full-length genomes generated in this study) (Supplementary Table S2). Given increased accessibility to the S gene sequences in the public domain, similar dating analysis was estimated using 114 complete and partial S gene sequences (Supplementary Table S3). To infer the mean tMRCA and the 95% highest posterior density (HPD), the exponential population size under a relaxed-clock model with BSP distribution and uncorrelated exponential distribution were adopted for the S gene and full-length genome, respectively. Both models were the best data-fitting coalescent models and were selected by means of marginal likelihoods (specifically Bayes factor), as estimated using the smoothed harmonic mean estimator^35^ and by means of Akaike's Information Criterion for MCMC samples estimated using the method-of-moments estimator implemented in Tracer (data not shown).^36^ The molecular clock dating analysis estimated the mean evolutionary rate (and 95% HPD) for the S gene and full-length genome of all HCoV-OC43 strains based on their respective coalescent models at 5.8 × 10^−4^ (4.4 × 10^−^^4^ to 7.1 × 10^−^^4^) and 1.8 × 10^−4^ (1.2 × 10^−4^ to 2.4 × 10^−^^4^) nucleotide substitutions per site per year, respectively. The estimate of the mean evolutionary rate for the S gene is comparable to previous findings of 6.1 × 10^−4^–6.7 × 10^−4^ nucleotide substitutions per site per year,^7, 20, 37^ whereas the rate for a full-length genome, to our knowledge, is newly estimated in this study. As shown in Table 2, the estimated tMRCA based on the S gene and full-length genome data for genotype A was in the 1960s, genotypes B to E were in the late 1990s to mid-2000s, and genotypes F and G were in the late 2000s to early 2010s. The estimates from both sets of data were comparable, indicating that the S gene or the full-length genome could be used for tMRCA estimation.

### HCOV-OC43 transmission network

**Figure 6:**
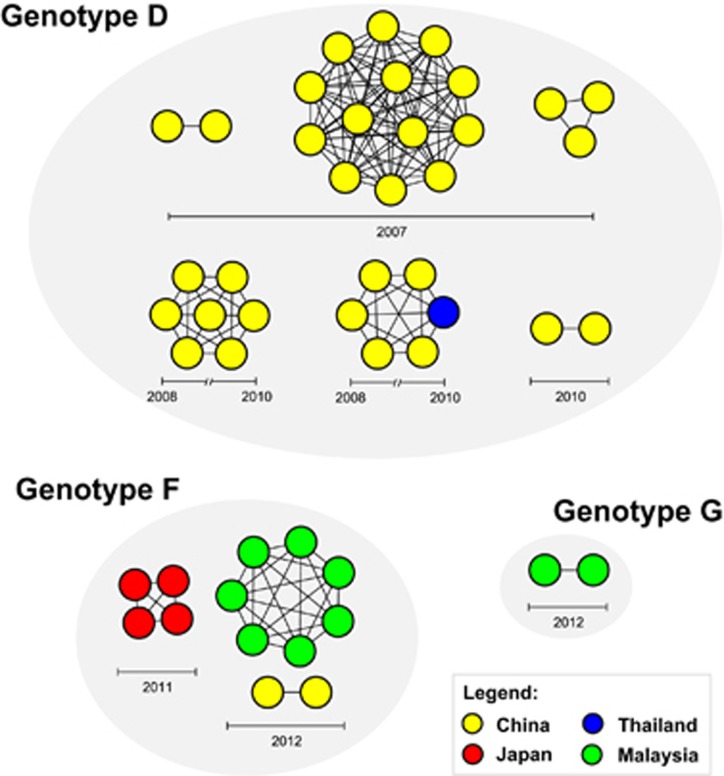
Transmission network of HCoV-OC43 genotypes D, F and G. Transmission clusters were inferred from 86 S gene sequences based on the Tamura-Nei 93 (TN93)^29^ pairwise distance estimates performed using a custom script in Python (release 3.2.6) with 1000 bootstrap replicates.

To investigate the transmission pattern of HCoV-OC43 genotypes D, F and G, transmission clusters were constructed based on the pairwise distances using the TN93 model estimated from 86S gene sequences, which included sequences from China, Japan, Thailand, France and Malaysia collected between 2002 and 2013 (Supplementary Table S3). The 95% confidence interval of the lower 0.025 percentile of the inter-person patristic distance was calculated at 0.001 substitutions per site, which represented the distance threshold for estimating HCoV-OC43 transmission cluster (Supplementary Table S4). Forty-eight sequences (55.8%, 48/86) formed a total of ten transmission clusters with strong spatial structure, of which four dyads and six networks of different sizes ranging between 3 and 13 nodes per network were estimated (Figure 6). For genotype D, five transmission clusters involved sequences that were isolated from China between 2007 and 2010, whereas one transmission network was shared among sequences from China and Thailand sampled within a 2-year period (2008–2010). However, three genotype F and one genotype G clusters were circulating exclusively within their particular countries of origin: Japan in 2011, China and Malaysia in 2012 (Figure 6).

## Discussion

HCoV-OC43 strains are associated with respiratory diseases and have caused outbreaks worldwide.^8, 19, 38, 39^ Despite its first discovery in 1967,^34^ full-length genomes of known and published HCoV-OC43 genotypes were limited. More recently, genotyping studies have become more common, with the first description of a complete genome from a laboratory ATCC strain and a clinical isolate from France in 2004 (genotype A)^34^ followed by two Belgium strains in 2005 (genotypes B and C).^5^ In the early 2010s, genotyping studies further highlighted the epidemiological impact of recombination in driving the emergence of two more novel HCoV-OC43 genotypes (genotypes D and E). Genotype D was a result of recombination between genotypes B and C,^7^ whereas genotype E was a recombinant among genotypes B, C and D.^9^

The global emergence and re-emergence of viral respiratory disease outbreaks have prompted more active pathogen surveillance initiatives in major healthcare settings worldwide, including in Southeast Asia. In Malaysia, a cross-sectional molecular surveillance of HCoV-OC43 was conducted among patients with acute URTI in a major teaching hospital in Kuala Lumpur.^20^ On the basis of the phylogenetic analysis of the S gene, two unique lineages (lineages 1 and 2) appeared to diverge from genotype D. HCoV-OC43 strains found in other geographical regions were also grouped within the two lineages. Phylogenetic incongruence found in the partial genes of these two unique lineages indicated possible recombination between genotypes, which prompted the sequencing of their complete genomes presented in this study.

Our analyses on the full-length genome sequences of the unique HCoV-OC43 strains from novel lineages 1 and 2^20^ confirm the identification of two novel genotypes, which are designated as genotypes F and G. These two novel genotypes were descendants of a previously reported recombinant genotype D,^7^ which contained genotypes B and C as the putative parental genotypes and a genotype A-like genetic signal in the ORF1b gene. The recombination breakpoints were located at ~16 000–17 000 nt and 17 000–17 500 nt, which corresponds to the nsp9/nsp10 junctions (Figure 2A). Previous studies on HCoV-OC43 genotypes D and E genomes have reported potential recombination sites at the nsp2/nsp3, nsp6/nsp7, nsp9/nsp10, nsp12/nsp13, ns2α/HE, ns5α/E and M/N junctions.^7, 9^ In addition, recombination breakpoints in the nsp5/nsp6, nsp16/S and nsp14/nsp15 junctions were also identified in other HCoV genomes, such as HCoV-HKU1,^15^ SARS-CoV^40^ and MERS-CoV,^41^ respectively. It is notable that the ORF1ab region (a region that encodes for non-structural proteins) is probably more recombination-prone compared with other regions in the HCoV genome. Recombination in this region typically contributes to the generation of new HCoV genotypes,^7, 9, 15, 40, 41^ suggesting the importance to target this region for molecular evolutionary and epidemiological investigations.

Apart from recombination analysis, genotypes F and G strains also shared four and eight nucleotide substitutions, respectively, with genotype A within the ORF1b, HE and S gene region (Figure 3). This finding implies that minute genotype A-like genetic signals found within these regions differentiate genotypes F and G; however, the breakpoints could not be clearly resolved through bootscan analysis. In addition to minor differences in the recombination pattern, the distinction of the genomes between genotypes F and G could be attributed to their possession of unique signature nucleotide and amino acid mutations across the full-length genomes. Most of the signature mutations found only in genotypes F or G occurred in the S gene (Figure 3), which is not uncommon given that the spike protein is a major antigenic surface protein that undergoes high selection pressure exerted by the host immune response.^4^ Three signature amino acid mutations at the spike protein, H177Y (genotype F), D267K and I268D (genotype G) (or H176Y, D266K and I267D according to Ren *et al.*,^11^ were among the positively selected sites identified at the N-terminal domain (NTD) of the S1 subunit. More interestingly, a three-nucleotide insertion (GGC), which resulted in the introduction of a glycine residue in the NTD, was unique only to the Malaysian genotype F strains. Whether the introduction of glycine has a role in enhancing the binding of NTD to the sugar receptors of the host cells, which subsequently enhances the pathogenicity of HCoV-OC43,^4^ requires further investigation.

In this study, evidence from phylogenetic, amino acid and recombination analyses was used to demonstrate the global distribution of HCoV-OC43 genotypes F and G. Due to limited published and genotyped full-length genome data in the database, a larger amount of S gene sequence data was utilized. Evidence from these analyses revealed that several strains that were previously classified as genotype D from China, Thailand, Japan and France belonged to genotypes F or G. Such misclassification highlights the potential weaknesses in the current classification system that relies primarily on the phylogenetic analysis of three different parts of the genome: S, RdRP (nsp12), and N genes. The use of these parts promotes bias towards misclassification of strains with recombination occurring outside these regions.^7, 9, 10, 19, 20, 42^ In addition, the highly conserved RdRP and N genes typically result in poorly resolved phylogenetic trees,^9, 10, 20^ which is not ideal for precise genotype classification. Therefore, to minimize the underestimation of recombinant strains, full-length genomes should be characterized for new genotype designation and shared in public databases as reference genotypes. As shown in Figure 1, the pairwise genetic distances of full-length genome sequences estimated between non-recombinant HCoV-OC43 genotypes (A vs. B, A vs. C, and B vs. C) were >0.6%, which is suggestive of a minimal genetic distance for a genotype assignment. Such a cutoff, however, is not applicable for recombinant lineages that contain genetic information from multiple parental genotypes with varying evolutionary histories. Similar to the more established nomenclature systems used for the human immunodeficiency virus type 1 and hepatitis C virus,^43, 44^ we propose that inter-genotype recombinant strains of HCoV-OC43 identified among multiple individuals can be assigned as ‘recombinant form' (RF) to reflect the recombination origin of the lineage. The RF candidates, which descended from the same parental genotypes via recombination event(s), must share an identical mosaic recombinant structure based on the full-length genome sequences. RF candidates are suffixed with an identifying number in the order in which they were first described (01, 02, 03 and so on) followed by letters (listed alphabetically), indicating the parental genotypes involved in the recombination. For example, HCoV-OC43 genotype D, which was the first described inter-genotype recombinant involving genotypes B and C, can be designated as RF01_BC. For clarity, if more than two parental genotypes are involved in the recombination, a complex (cpx) recombinant lineage can be assigned. Therefore, in the case of genotype E and the newly described genotypes F and G, they can be (re)designated as RF02_cpx, RF03_cpx and RF04_cpx, respectively. The newly proposed nomenclature system for non-recombinant and recombinant HCoV-OC43 herein will establish a platform for a more consistent and reliable naming system that considers genetic recombination as an important and common feature in HCoV evolution. The application of a naming system that reflects the evolutionary histories of HCoV-OC43 strains will also enable better mapping and tracking of newly emerging recombinant lineages, which have become increasingly prevalent in large parts of Asia and elsewhere.^7, 9, 20^

Communicable diseases, such as virus-associated respiratory infections, are transmitted through close contacts between individuals within networks. The role of networks in fueling and sustaining the onward transmission of pathogen is profound,^45^ unless effective intervention measures are being introduced. The inclusion of transmission clusters information coupled with epidemiological surveillance data could help to better understand the spread and dynamics of viral diseases attributed to specific genotypes circulating in the population. To the best of our knowledge, such analysis represents an inventive approach of which the spread and dynamics of HCoV-OC43 transmission was mapped. Using the TN93 pairwise distance estimates of the S gene to establish genetically-related HCoV-OC43 strains with apparent transmission linkage, we inferred the transmission clusters of genotypes D, F and G sampled across Asia and Europe between 2002 and 2013. Greater than 55% of the global spread of genotype D were linked to transmission clusters, in which 90% of the transmissions were restricted within China, except for one genotype D network that involved sequences from China and Thailand, suggesting cross-border transmission and persistence of a transmission network for up to a 2-year period (Figure 6). In addition, the emergence and persistence of multiple sub-epidemic clusters of genotype D corroborates with the recent predominance and continual transmission of this genotype in China.^9, 11^

In summary, herein we report two novel HCoV-OC43 recombinant genotypes, designated as genotypes F and G, which were identified among patients presenting with acute respiratory tract symptoms. Using observations from phylogenetic, recombination, and comparative genomic analyses on full-length genome sequences, HCoV-OC43 genotypes F and G were likely to co-circulate worldwide. Bayesian coalescent dating analysis implied that both genotypes probably diverged concurrently around the late 2000s to early 2010s from a genotype D-like common ancestor through natural recombination a few years after genotype D was first identified in Asia. The wide distribution of HCoV-OC43 genotype D and its recombinant lineages were driven in part by the emergence and persistence of transmission networks in this region. Overall, our results highlight the importance of recombination in HCoV-OC43 evolution, which warrants a more consistent nomenclature system for classification and better tracking of newly emerged recombinant lineages. More comprehensive molecular surveillance studies on HCoV-OC43 are essential to better understand the evolutionary dynamics, pathogenesis, and disease burden of HCoV-OC43 infections.

## Figures and Tables

**Table 1:** Demographic and clinical profile of patients infected with HCoV-OC43 lineages 1 and 2

**Lineage**	**Strain ID**	**Collection date**	**Demographic profile**		**Symptoms reported**
			**Age**	**Sex**	
Lineage 1	MY-U208/12	28 March 2012	61	F	Sneezing, nasal discharge, sore throat, hoarseness of voice, cough
	MY-U413/12	2 May 2012	72	F	Nasal discharge
	MY-U464/12	9 May 2012	38	M	Nasal congestion, headache, sore throat, hoarseness of voice, cough
	MY-U523/12	18 May 2012	74	M	Sneezing, cough
	MY-U732/12	25 June 2012	53	F	Sneezing, nasal discharge, headache, cough
	MY-U868/12	16 July 2012	59	M	Nasal congestion, cough
	MY-U945/12	1 August 2012	11	M	Sneezing, nasal discharge, nasal congestion, headache
	MY-U1024/12	24 August 2012	61	F	Nasal congestion
	MY-U1140/12	10 September 2012	21	M	Sneezing, nasal discharge, sore throat, hoarseness of voice
Lineage 2	MY-U002/12	22 February 2012	71	F	Nasal congestion, headache, cough
	MY-U236/12	2 April 2012	19	M	Nasal discharge, nasal congestion, headache
	MY-U710/12	20 June 2012	50	F	Sneezing, nasal discharge, nasal congestion, cough
	MY-U774/12	2 July 2012	32	F	Sneezing, nasal congestion, headache, sore throat, hoarseness of voice, cough
	MY-U1057/12	27 August 2012	58	F	Sore throat, hoarseness of voice, cough
	MY-U1758/13	2 January 2013	56	M	Sneezing, nasal discharge, nasal congestion, headache, sore throat, cough
	MY-U1975/13	15 February 2013	52	F	Sneezing, headache, hoarseness of voice, cough

Abbreviations: Female, F; Male, M.

**Table 2:** Time of most recent common ancestor (tMRCA) for HCoV-OC43 genotypes A to G estimated based on the spike (S) gene and full-length genome

**Genotype**	**tMRCA (95% HPD)**	
	**Spike (S) gene**	**Full-length genome**
A	1963.8 (1959.4–1966.7)	1964.7 (1960.3–1966.8)
B	1999.1 (1996.6–2001.5)	1999.0 (1993.5–2002.6)
C	1997.2 (1994.0–1999.7)	1998.0 (1994.3–2000.5)
D	1997.3 (1994.2–2000.4)	1999.3 (1996.5–2001.5)
E	2007.9 (2005.9–2009.4)	2003.3 (1999.1–2006.9)
F	2010.8 (2010.0–2011.4)	2009.8 (2008.1–2011.2)
G	2010.7 (2010.0–2011.4)	2010.1 (2008.5–2011.4)

Abbreviation: highest posterior density, HPD.

## Supplementary Material

Supplementary Figure 1

Supplementary Figure 1 (Continued)
