# Supplementary material for: Identification and evolutionary dynamics of two novel human coronavirus OC43 genotypes associated with acute respiratory infections: phylogenetic, spatiotemporal and transmission network analyses
Source: Emerg Microbes Infect. 2017 Jan 4;6(1):e3–. doi: 10.1038/emi.2016.132 (PMC5285497; doi:10.1038/emi.2016.132)
Supplement: Supplementary Figure 1 [file emi2016132x1.pdf]

Supplementary Figure S1

A) NJ Tree

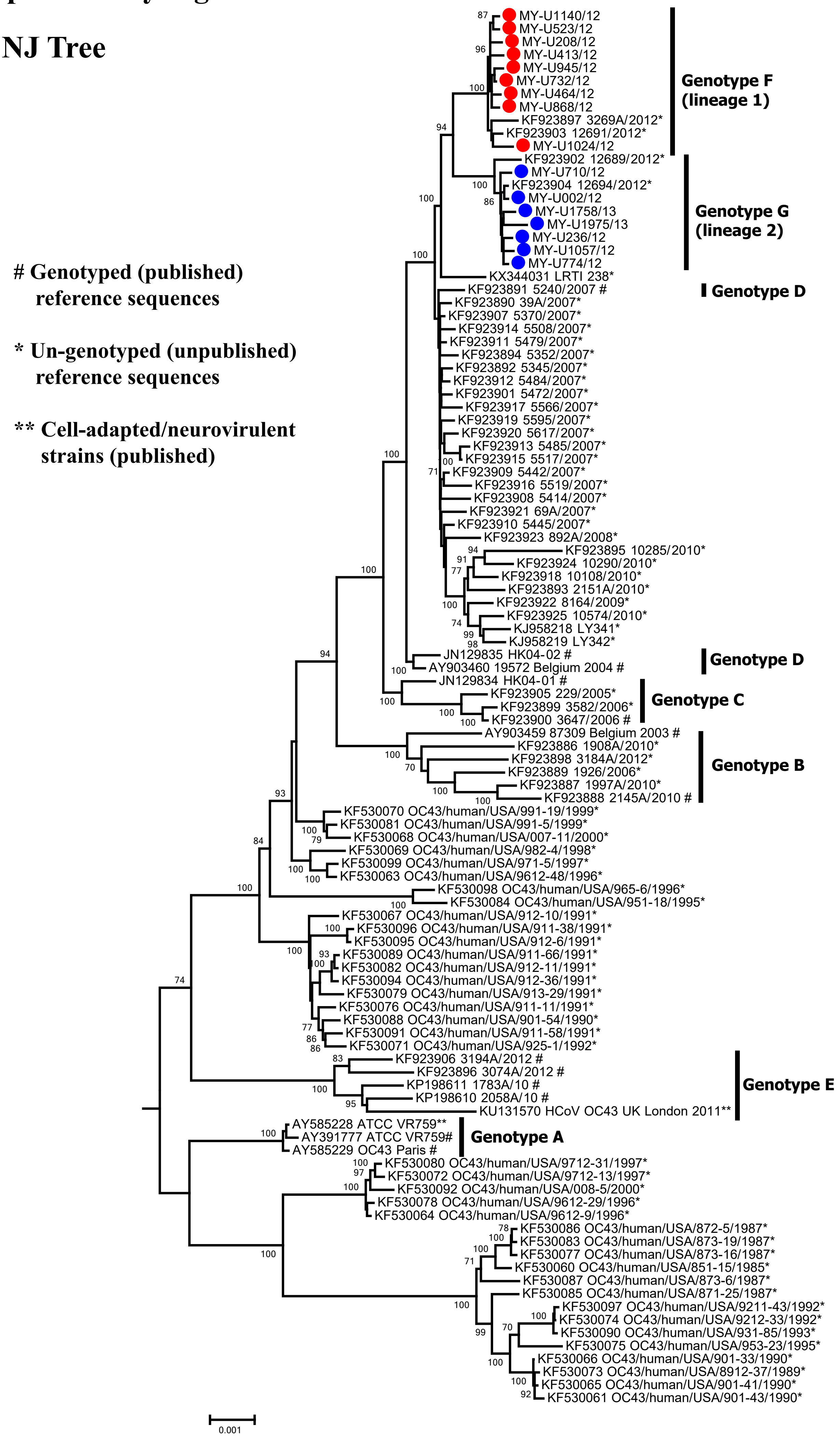

B) ML Tree

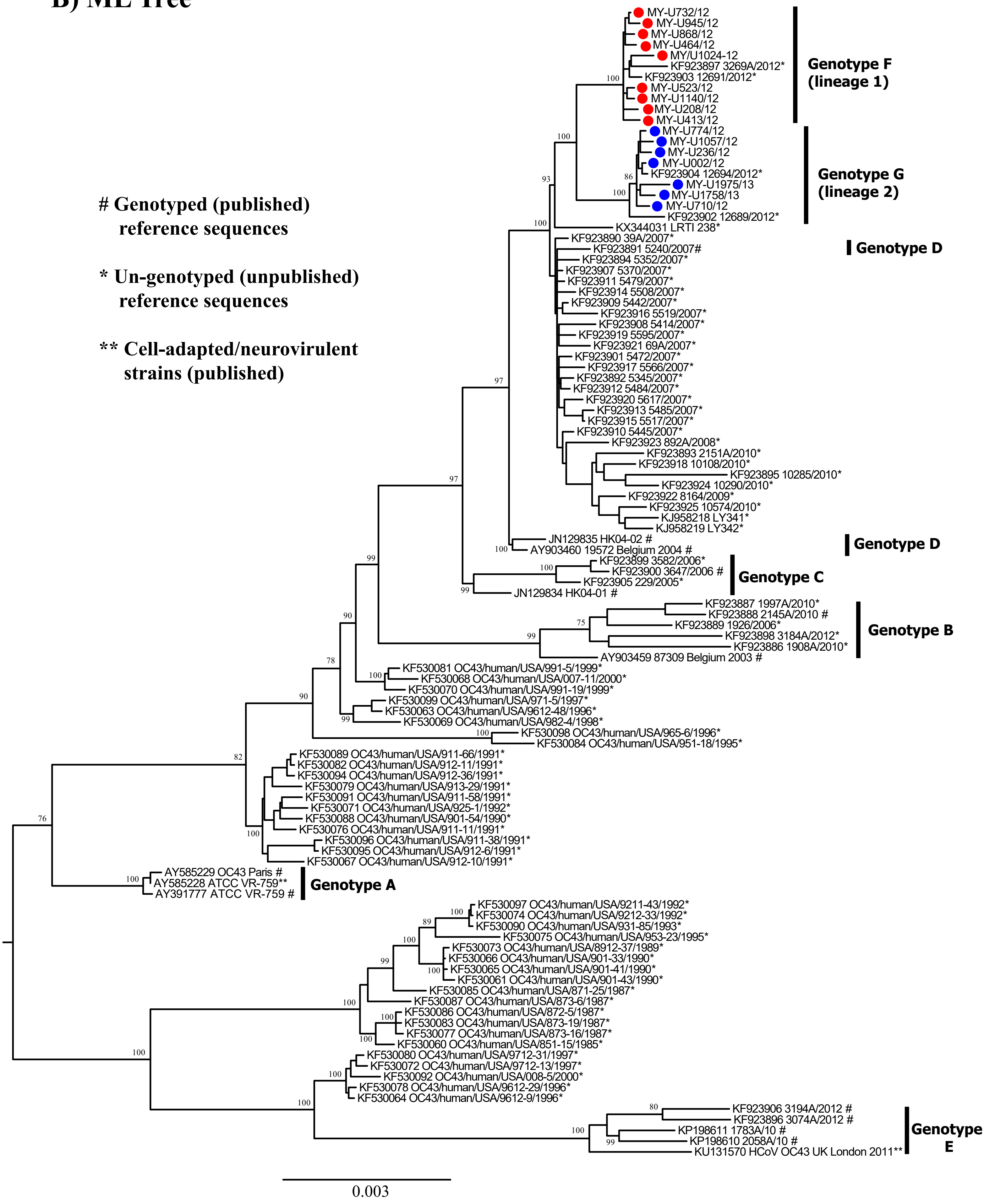

Genotyped (published) reference sequences
* Un-genotyped (unpublished) reference sequences
** Cell-adapted/neurovirulent strains (published)
MY-U732/12
MY-U945/12
MY-U868/12
MY-U464/12
MY/U1024-12
KF923897 3269A/2012*
KF923903 12691/2012*
MY-U523/12
MY-U1140/12
MY-U208/12
MY-U413/12
Genotype F (lineage 1)
MY-U774/12
MY-U1057/12
MY-U236/12
MY-U002/12
KF923904 12694/2012*
MY-U1975/13
MY-U1758/13
MY-U710/12
KF923902 12689/2012*
Genotype G (lineage 2)
KX344031 LRTI 238*
KF923890 39A/2007*
KF923891 5240/2007#
Genotype D
KF923894 5352/2007*
KF923907 5370/2007*
KF923911 5479/2007*
KF923914 5508/2007*
KF923909 5442/2007*
KF923916 5519/2007*
KF923908 5414/2007*
KF923919 5595/2007*
KF923921 69A/2007*
KF923901 5472/2007*
KF923917 5566/2007*
KF923892 5345/2007*
KF923912 5484/2007*
KF923920 5617/2007*
KF923913 5485/2007*
KF923915 5517/2007*
KF923910 5445/2007*
KF923923 892A/2008*
KF923893 2151A/2010*
KF923918 10108/2010*
KF923895 10285/2010*
KF923924 10290/2010*
KF923922 8164/2009*
KF923925 10574/2010*
KJ958218 LY341*
KJ958219 LY342*
JN129835 HK04-02 #
AY903460 19572 Belgium 2004 #
Genotype D
KF923899 3582/2006*
KF923900 3647/2006 #
KF923905 229/2005*
JN129834 HK04-01 #
Genotype C
KF923887 1997A/2010*
KF923888 2145A/2010 #
KF923889 1926/2006*
KF923898 3184A/2012*
KF923886 1908A/2010*
AY903459 87309 Belgium 2003 #
Genotype B
KF530081 OC43/human/USA/991-5/1999*
KF530068 OC43/human/USA/007-11/2000*
KF530070 OC43/human/USA/991-19/1999*
KF530099 OC43/human/USA/971-5/1997*
KF530063 OC43/human/USA/9612-48/1996*
KF530069 OC43/human/USA/982-4/1998*
KF530098 OC43/human/USA/965-6/1996*
KF530084 OC43/human/USA/951-18/1995*
KF530089 OC43/human/USA/911-66/1991*
KF530082 OC43/human/USA/912-11/1991*
KF530094 OC43/human/USA/912-36/1991*
KF530079 OC43/human/USA/913-29/1991*
KF530091 OC43/human/USA/911-58/1991*
KF530071 OC43/human/USA/925-1/1992*
KF530088 OC43/human/USA/901-54/1990*
KF530076 OC43/human/USA/911-11/1991*
KF530096 OC43/human/USA/911-38/1991*
KF530095 OC43/human/USA/912-6/1991*
KF530067 OC43/human/USA/912-10/1991*
AY585229 OC43 Paris #
AY585228 ATCC VR-759**
AY391777 ATCC VR-759 #
Genotype A
KF530097 OC43/human/USA/9211-43/1992*
KF530074 OC43/human/USA/9212-33/1992*
KF530090 OC43/human/USA/931-85/1993*
KF530075 OC43/human/USA/953-23/1995*
KF530073 OC43/human/USA/8912-37/1989*
KF530066 OC43/human/USA/901-33/1990*
KF530065 OC43/human/USA/901-41/1990*
KF530061 OC43/human/USA/901-43/1990*
KF530085 OC43/human/USA/871-25/1987*
KF530087 OC43/human/USA/873-6/1987*
KF530086 OC43/human/USA/872-5/1987*
KF530083 OC43/human/USA/873-19/1987*
KF530077 OC43/human/USA/873-16/1987*
KF530060 OC43/human/USA/851-15/1985*
KF530080 OC43/human/USA/9712-31/1997*
KF530072 OC43/human/USA/9712-13/1997*
KF530092 OC43/human/USA/008-5/2000*
KF530078 OC43/human/USA/9612-29/1996*
KF530064 OC43/human/USA/9612-9/1996*
KF923906 3194A/2012 #
KF923896 3074A/2012 #
KP198611 1783A/10 #
KP198610 2058A/10 #
KU131570 HCoV OC43 UK London 2011**
Genotype E
0.003
