# Supplementary figures and images for: Identification and evolutionary dynamics of two novel human coronavirus OC43 genotypes associated with acute respiratory infections: phylogenetic, spatiotemporal and transmission network analyses
Source: Emerg Microbes Infect. 2017 Jan 4;6(1):e3–. doi: 10.1038/emi.2016.132 (PMC5285497; doi:10.1038/emi.2016.132)

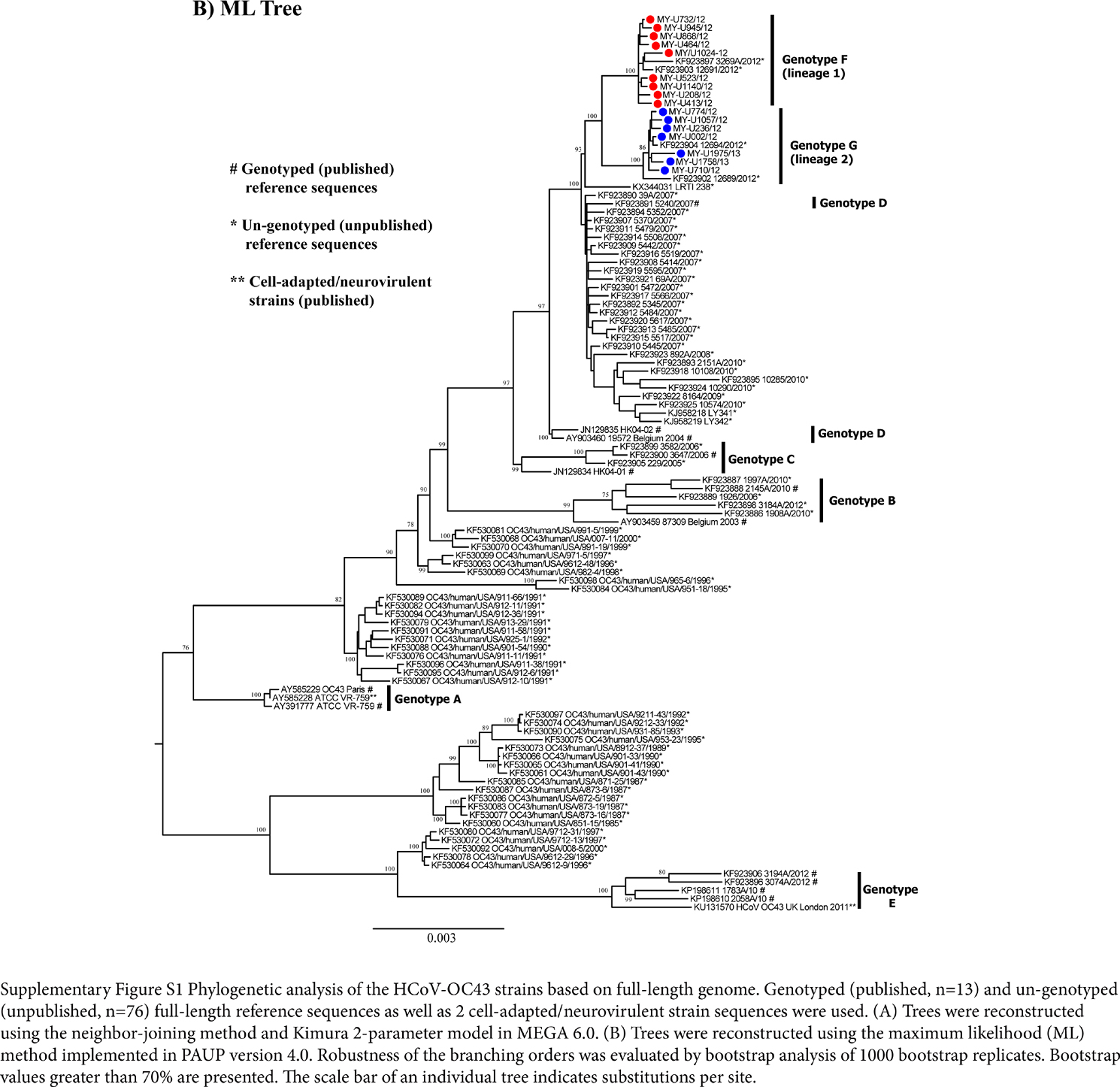

Supplement: Supplementary Figure 1 (Continued) [file emi2016132x2.tif]
