# Supplementary material for: Identification and evolutionary dynamics of two novel human coronavirus OC43 genotypes associated with acute respiratory infections: phylogenetic, spatiotemporal and transmission network analyses
Source: Emerg Microbes Infect. 2017 Jan 4;6(1):e3–. doi: 10.1038/emi.2016.132 (PMC5285497; doi:10.1038/emi.2016.132)
Supplement: Supplementary Table 1 [file emi2016132x3.docx]

**Supplementary Table S1** Primers used for full-length genome sequencing of HCoV-OC43

| **Fragment** | **Target Gene** | **Primers (1)** | **Primer (5' - 3')** | **Primer Length** | **Product Length** |
| --- | --- | --- | --- | --- | --- |
| 1 | ORF1a | OC43F1 | **GATTGTGAGCGATTTGC** | 17 | 665 |
|  |  | OC43R665 | **TAAGCATGTGGCCTATCAGTT** | 21 |  |
| 2 |  | OC43F557 | **ATGCTATGTGAGAGGTTGTAATCC** | 24 | 977 |
|  |  | OC43R1531 | **CCTGTTAAYCCTGTATTGCATA** | 22 |  |
| 3 |  | OC43F1410 | **GGTTGGATTCCTGGTAACATGA** | 22 | 1026 |
|  |  | OC43R2431 | **GAATTTGTCTTTCAGGCAGAAAA** | 23 |  |
| 4 |  | OC43F2317 | **TTGCACAAGCATTTCAGAGTGTT** | 23 | 1092 |
|  |  | OC43R3396 | **GTTAAAGTTTTGGGTCTGTATGTGC** | 25 |  |
| 5 |  | OC43F3279 | **GATGTAGAAATGTCGGATTTTG** | 22 | 1128 |
|  |  | OC43R4405 | **TGCCTTCTGATGTGTCTTTA** | 20 |  |
| 6 |  | OC43F4293 | **AGAACACAGGGTAAACAAAGTT** | 22 | 1243 |
|  |  | OC43R5523 | **ACATTGAGTCGTGARGATCTTG** | 22 |  |
| 7 |  | OC43F5450 | **TTGCATGTAAATGTGGTGTAA** | 21 | 584 |
|  |  | OC43R6033 | **TAATKCTAAGTTGGGTTTTGAT** | 22 |  |
| 8 |  | OC43F5949 | **CGAGAAAGTAGATGGTGTGTAT** | 22 | 666 |
|  |  | OC43R6614 | **AATGTGGTTAAAGCTGTGCG** | 20 |  |
| 9 |  | OC43F6426 | **TGATGTGTATGATATGTGGCTTA** | 23 | 509 |
|  |  | OC43R6934 | **TAAGATTGCACAGTGGATTA** | 20 |  |
| 10 |  | OC43F6472 | **TGAGCAGAGCAGTTAACGTACCTA** | 24 | 704 |
|  |  | OC43R7177 | **TGTATACGGCATGGTTTTATCCA** | 23 |  |
| 11 |  | OC43F7090 | **GAAGCTGATAGGAGAGCATT** | 20 | 1115 |
|  |  | OC43R8204 | **TAACTTGGTGCCAACATATTT** | 21 |  |
| 12 |  | OC43F8068 | **CGTAAAAGTTGTTCTATTGATTCAG** | 25 | 334 |
|  |  | OC43R8400 | **CTGAAGCTTACTTATAATAAGCAGA** | 25 |  |
| 13 |  | OC43F8304 | **TATATGGTCTGTGGATGCTTT** | 21 | 607 |
|  |  | OC43R8910 | **ATGCTAGTGGCTGTGTGCTT** | 20 |  |
| 14 |  | OC43F8775 | **CCCTACCAAAGTGTTACGATA** | 21 | 653 |
|  |  | OC43R9427 | **TAAYGTGATTGTGTGGTGTG** | 20 |  |
| 15 |  | OC43F9314 | **CTGGTGCTATACTCGCTGTAA** | 21 | 612 |
|  |  | OC43R9925 | **TTACCAACCKCCTACWGCTT** | 20 |  |
| 16 |  | OC43F9844 | **GCTTGCTCTCAGTTGGCTAA** | 20 | 641 |
|  |  | OC43R10484 | **ATACTGGTACTGACTTCAATGG** | 22 |  |
| 17 |  | OC43F10297 | **AAACCACAAGGAGCYTTTCATGTAA** | 25 | 482 |
|  |  | OC43R10780 | **CCAAGGACGTCAGATTATGGGTAG** | 24 |  |
| 18 |  | OC43F10656 | **CAATGGATTTAGCCAAGTTA** | 20 | 780 |
|  |  | OC43R11435 | **ATTGCTAAGTGGGTTGCTGT** | 20 |  |
| 19 |  | OC43SF11302 | **GGTACAAGGGTTCTAACTTAGAGG** | 24 | 823 |
|  |  | OC43SR12124 | **CCTGTAATATTGCTAAATCTGCTTA** | 25 |  |
| 20 |  | OC43F12021 | **CGTTGAATATGAAGTTGCTAA** | 21 | 419 |
|  |  | OC43R12439 | **AATRTCTATGTTACCTATGCGG** | 22 |  |
| 21 |  | OC43F12318 | **TGTGTACCATTGAATGCAATA** | 21 | 487 |
|  |  | OC43R12804 | **CTGTTCAAGATGCTAAAGGTC** | 21 |  |
| 22 |  | OC43F12703 | **CTTAGTGATGTTGATGGTCTTA** | 22 | 583 |
|  |  | OC43R13285 | **ATTTTGGCGGGATGGAAGTT** | 20 |  |
| 23 | RdRp | OC43F13180 | **AGTTTGTACAAGTGCCTGTAGGT** | 23 | 1122 |
|  |  | OC43R14301 | **CTTGTTAGGCAAATTTTTGTGGA** | 23 |  |
| 24 |  | OC43F14160 | **GGAGTATGCCATATCATCCTAACAC** | 25 | 1029 |
|  |  | OC43R15162 | **GCTATGCCAAACMTACTACGYATTG** | 25 |  |
| 25 |  | LPW_3064F | **CTGGGATGATATGTTACGCCG** | 21 | 660 |
|  |  | LPW_2579R | **CAYGARTTYTGTTCACAACACAC** | 23 |  |
| 26 |  | LPW_1223F | **TAAGTGCCTTTCAACAGGT** | 19 | 881 |
|  |  | LPW_1127R | **GCAGAAACGCAAAAGGCA** | 18 |  |
| 27 | ORF1b | OC43F16382 | **ACAGGATCTCCGTACATAGACGA** | 23 | 1081 |
|  |  | OC43R17471 | **GTCCGCCTTGGTTTATGAAA** | 20 |  |
| 28 |  | OC43F17315 | **GCACCACGTGTGTTATTGAG** | 20 | 1031 |
|  |  | OC43R18345 | **TCCTCCTGGTGAACAATTTAA** | 21 |  |
| 29 |  | OC43F18174 | **GTGCTCATGCCACGCKTGATA** | 21 | 1089 |
|  |  | OC43R19271 | **TGTGTGTATATGGATGGYATGGATGCTA** | 28 |  |
| 30 |  | OC43F19059 | **CACCGAATGCAGTTGTATGTAGAT** | 24 | 1089 |
|  |  | OC43R20157 | **CTATCTTTACACAAAGCCGTGTTA** | 24 |  |
| 31 |  | OC43F19994 | **GTGCGTAAAGAAGGTCAGGATGT** | 23 | 886 |
|  |  | OC43R20879 | **GGTTGCCTGCTGGTACTATTC** | 21 |  |
| 32 |  | OC43F20689 | **GAAGCCAGTTACTTTGCCTACAG** | 23 | 1003 |
|  |  | OC43R21691 | **GGAYATACAGCCTGAAGACTATAAA** | 25 |  |
| 33 | NS2α | OC43F21507 | **ATGCAGACAAGCCTAATCATTTTAT** | 25 | 757 |
|  |  | OC43R22266 | **CTACAGGAAAAGTTTTCATTTCCG** | 24 |  |
| 34 | HE | OC43HE22158F | **AGCTAGAAGAAGGCGATCTTCC** | 22 | 902 |
|  |  | OC43HE23056R | **GACACTGGTGTTATYTATGGTCTCA** | 25 |  |
| 35 |  | OC43HE22867F | **CCTGCWTATATAGCTCCTCRAGC** | 23 | 898 |
|  |  | OC43HE23770R | **CGGTCCTCCTYCTATAAGTACTGM** | 24 |  |
| 36 | Spike | LPW_1162F | **CCYRTTTGTRTGTATGATCC** | 20 | 824 |
|  |  | LPW_1166R | **GGTGGTACYTTTTATGCA** | 18 |  |
| 37 |  | LPW_1261F | **CTGCYATARYTATAGGTAGT** | 20 | 890 |
|  |  | LPW_2094R | **CCTACAATTGGGYAATTTGGGC** | 22 |  |
| 38 |  | LPW_2095F | **TGATGCTGCTAAGATATATGG** | 21 | 952 |
|  |  | LPW_2098R | **CCAGCATTGCTATTTCGGAAT** | 21 |  |
| 39 |  | LPW_1839F | **ACCTTTTATATGATTCTAATGG** | 22 | 1043 |
|  |  | LPW_1178R | **ATTAATGGKCTTGGTGTC** | 18 |  |
| 40 |  | OC43F26457 | **GACCTCATTTGTGTGCAAAG** | 20 | 886 |
|  |  | OC43R27323 | **CACTTCAAYACCCAACCTTC** | 20 |  |
| 41 |  | OC43F27204 | **ATACTTGGATGTACACTGGTAGTG** | 24 | 637 |
|  |  | OC43R27806 | **TGAGAAGAAATWTCTCCGTTATAT** | 24 |  |
| 42 | NS5α, E, M | OC43F27758 | **TCCAATCTAGCATTTGTTACCACG** | 24 | 1232 |
|  |  | OC43R28982 | **AAGCGATACTAGTGGTTTTGCTGTT** | 25 |  |
| 43 | M, N | OC43F28865 | **ACTAGGTAYTGGCTATTCTTTGGC** | 24 | 966 |
|  |  | OC43R29830 | **GCAAAACTTGGCAAGGATGC** | 20 |  |
| 44 | N | OC43F29639 | **GGGTTACTATATTGAAGGCTCAGG** | 24 | 1084 |
|  |  | OC43R74 | **TGGAAGAATCACAAAAAAAAAA** | 22 |  |
| Walking |  | 3’raceF | **AATGGATKTCTTGCTGCTAT** | 20 | - |
|  |  | 5’raceR392 | **ATTGTCGCCGACTTCTTAAA** | 20 | - |

* Modification made to existing published primers is color-coded.

**Reference**

1. Zhang Y, Li J, Xiao Y *et al*. Genotype shift in human coronavirus OC43 and emergence of a novel genotype by natural recombination. *J Infect* 2015; **70**: 641-650.
