# Supplementary material for: Identification and evolutionary dynamics of two novel human coronavirus OC43 genotypes associated with acute respiratory infections: phylogenetic, spatiotemporal and transmission network analyses
Source: Emerg Microbes Infect. 2017 Jan 4;6(1):e3–. doi: 10.1038/emi.2016.132 (PMC5285497; doi:10.1038/emi.2016.132)
Supplement: Supplementary Table 2 [file emi2016132x4.docx]

**Supplementary Table S2** Background information for published and genotyped global full-length genome of HCoV-OC43 strains used for phylogenetic analysis

| Strain Name | Genotype | Accession Number | Sampling Year | Isolation Sites | Reference |
| --- | --- | --- | --- | --- | --- |
| ATCC VR-759 | A | AY391777 | 1960s | United Kingdom | (1) |
| HCoV-OC43-Paris | A | AY585229 | 2001 | Paris, France | (2) |
| 87309 Belgium 2003 | B | AY903459 | 2003 | Belgium | (3) |
| 2145A/2010 | B | KF923888 | 2010 | Beijing, China | (4) |
| HK04-01 | C | JN129834 | 2004 | Hong Kong, China | (5) |
| 3647/2006 | C | KF923900 | 2006 | Beijing, China | (4) |
| 19572 Belgium 2004 | D | AY903460 | 2004 | Belgium | (3) |
| HK04-02 | D | JN129835 | 2004 | Hong Kong, China | (5) |
| 5240/2007 | D | KF923891 | 2007 | Beijing, China | (4) |
| 3074A/2012 | E | KF923896 | 2012 | Beijing, China | (4) |
| 3194A/2012 | E | KF923906 | 2012 | Beijing, China | (4) |
| 2058A/10 | E | KP198610 | 2010 | Beijing, China | (4) |
| 1783A/10 | E | KP198611 | 2010 | Beijing, China | (4) |

**References**

1. Vijgen L, Keyaerts E, Moës E *et al*. Complete genomic sequence of human coronavirus OC43: molecular clock analysis suggests a relatively recent zoonotic coronavirus transmission event. *J Virol* 2005; **79**: 1595-1604.

2. St-Jean JR, Jacomy H, Desforges M, Vabret A, Freymuth F, Talbot PJ. Human respiratory coronavirus OC43: genetic stability and neuroinvasion. *J Virol* 2004; **78**:8824-8834.

3. Vijgen L, Keyaerts E, Lemey P *et al*. Circulation of genetically distinct contemporary human coronavirus OC43 strains. *Virology* 2005; **337**: 85-92.

4. Zhang Y, Li J, Xiao Y *et al*. Genotype shift in human coronavirus OC43 and emergence of a novel genotype by natural recombination. *J Infect* 2015; **70**: 641-650.

5. Lau SK, Lee P, Tsang AK *et al*. Molecular epidemiology of human coronavirus OC43 reveals evolution of different genotypes over time and recent emergence of a novel genotype due to natural recombination. *J Virol* 2011; **85**: 11325-11337.
