# Supplementary material for: Identification and evolutionary dynamics of two novel human coronavirus OC43 genotypes associated with acute respiratory infections: phylogenetic, spatiotemporal and transmission network analyses
Source: Emerg Microbes Infect. 2017 Jan 4;6(1):e3–. doi: 10.1038/emi.2016.132 (PMC5285497; doi:10.1038/emi.2016.132)
Supplement: Supplementary Table 3 [file emi2016132x5.docx]

**Supplementary Table S3** Information on published global HCoV-OC43 spike (S) gene sequences (complete and partial) used for phylogenetic analysis

| No | Strain Name | Genotype | Accession Number | Sampling Year | Isolation Sites | Length |
| --- | --- | --- | --- | --- | --- | --- |
| 1 | ATCC VR759 | A | AY391777 | 1960s | United Kingdom (1) | complete |
| 2 | HCoV-OC43-Paris | A | AY585229 | 2001 | Paris (2) | complete |
| 3 | 87309 Belgium 2003* | B | AY903459 | 2003 | Belgium (3) | complete |
| 4 | HCoV-OC43/FRA/EPI/Caen/2003/05* | B | KF963234 | 2003 | France (4) | complete |
| 5 | HCoV-OC43/FRA/EPI/Caen/2006/08* | B | KF963237 | 2006 | France | complete |
| 6 | 1926/06 | B | KF572807 | 2006 | Beijing, China (5) | complete |
| 7 | HCoV-OC43/FRA/EPI/Caen/2007/09* | B | KF963238 | 2007 | France | complete |
| 8 | HCoV-OC43/FRA/EPI/Caen/2008/10* | B | KF963239 | 2008 | France | complete |
| 9 | HCoV-OC43/FRA/EPI/Caen/2010/12* | B | KF963241 | 2010 | France | complete |
| 10 | 2145A/10 | B | KF572810 | 2010 | Beijing, China | complete |
| 11 | 1997A/10 | B | KF572808 | 2010 | Beijing, China | complete |
| 12 | 1919A/10 | B | KF572806 | 2010 | Beijing, China | complete |
| 13 | 1908A/10 | B | KF572805 | 2010 | Beijing, China | complete |
| 14 | 3184A/12* | B | KF572813 | 2012 | Beijing, China | complete |
| 15 | HCoV-OC43/Niigata.JPN/11-400 | B | AB695081 | 2011 | Japan (6) | partial |
| 16 | HCoV-OC43/Niigata.JPN/11-343 | B | AB695080 | 2011 | Japan | partial |
| 17 | HCoV-OC43/Niigata.JPN/11-335 | B | AB695079 | 2011 | Japan | partial |
| 18 | HCoV-OC43/Niigata.JPN/11-286* | B | AB695078 | 2011 | Japan | partial |
| 19 | HCoV-OC43/FRA/EPI/Caen/2001/02* | C | KF963231 | 2001 | France | complete |
| 20 | HK04-01* | C | JN129834 | 2004 | Hong Kong, China (7) | complete |
| 21 | 229/05* | C | KF572816 | 2005 | Beijing, China | complete |
| 22 | 3647/06* | C | KF572818 | 2006 | Beijing, China | complete |
| 23 | 3582/06 | C | KF572817 | 2006 | Beijing, China | complete |
| 24 | CU-1772/2010 | D | JX513268 | 2010 | Thailand (8) | partial |
| 25 | CU-H1187/2010* | D | JX513260 | 2010 | Thailand | partial |
| 26 | HCoV-OC43/Niigata.JPN/11-564* | D | AB695082 | 2011 | Japan | partial |
| 27 | HCoV-OC43/FRA/EPI/Caen/2002/03* | D | KF963232 | 2002 | France | complete |
| 28 | HK04-02* | D | JN129835 | 2004 | Hong Kong, China | complete |
| 29 | 19572 Belgium 2004* | D | AY903460 | 2004 | Belgium | complete |
| 30 | HCoV-OC43/FRA/EPI/Caen/2004/06* | D | KF963235 | 2004 | France | complete |
| 31 | 079A/07 | D | KF572820 | 2007 | Beijing, China | complete |
| 32 | 039A/07 | D | KF572819 | 2007 | Beijing, China | complete |
| 33 | 69A/07 | D | KF572864 | 2007 | Beijing, China | complete |
| 34 | 5656/07 | D | KF572863 | 2007 | Beijing, China | complete |
| 35 | 5625/07 | D | KF572862 | 2007 | Beijing, China | complete |
| 36 | 5617/07 | D | KF572861 | 2007 | Beijing, China | complete |
| 37 | 5595/07 | D | KF572860 | 2007 | Beijing, China | complete |
| 38 | 5566/07 | D | KF572859 | 2007 | Beijing, China | complete |
| 39 | 5519/07 | D | KF572858 | 2007 | Beijing, China | complete |
| 40 | 5517/07 | D | KF572857 | 2007 | Beijing, China | complete |
| 41 | 5508/07* | D | KF572856 | 2007 | Beijing, China | complete |
| 42 | 5485/07 | D | KF572855 | 2007 | Beijing, China | complete |
| 43 | 5484/07 | D | KF572854 | 2007 | Beijing, China | complete |
| 44 | 5479/07 | D | KF572853 | 2007 | Beijing, China | complete |
| 45 | 5472/07 | D | KF572852 | 2007 | Beijing, China | complete |
| 46 | 5445/07 | D | KF572851 | 2007 | Beijing, China | complete |
| 47 | 5442/07 | D | KF572850 | 2007 | Beijing, China | complete |
| 48 | 5414/07 | D | KF572849 | 2007 | Beijing, China | complete |
| 49 | 5370/07 | D | KF572848 | 2007 | Beijing, China | complete |
| 50 | 5352/07 | D | KF572847 | 2007 | Beijing, China | complete |
| 51 | 5345/07 | D | KF572846 | 2007 | Beijing, China | complete |
| 52 | 5331/07 | D | KF572845 | 2007 | Beijing, China | complete |
| 53 | 5240/07 | D | KF572844 | 2007 | Beijing, China | complete |
| 54 | 4954/07 | D | KF572843 | 2007 | Beijing, China | complete |
| 55 | 4795/07 | D | KF572842 | 2007 | Beijing, China | complete |
| 56 | 1034A/08 | D | KF572824 | 2008 | Beijing, China | complete |
| 57 | 1216A/08 | D | KF572830 | 2008 | Beijing, China | complete |
| 58 | 1157A/08 | D | KF572828 | 2008 | Beijing, China | complete |
| 59 | 1135A/08 | D | KF572827 | 2008 | Beijing, China | complete |
| 60 | 1081A/08 | D | KF572826 | 2008 | Beijing, China | complete |
| 61 | 892A/08 | D | KF572868 | 2008 | Beijing, China | complete |
| 62 | 978A/08* | D | KF572872 | 2008 | Beijing, China | complete |
| 63 | 9138/09 | D | KF572871 | 2009 | Beijing, China | complete |
| 64 | 9001/09 | D | KF572870 | 2009 | Beijing, China | complete |
| 65 | 8375/09 | D | KF572867 | 2009 | Beijing, China | complete |
| 66 | 8164/09* | D | KF572866 | 2009 | Beijing, China | complete |
| 67 | 8099/09 | D | KF572865 | 2009 | Beijing, China | complete |
| 68 | 1593A/09 | D | KF572837 | 2009 | Beijing, China | complete |
| 69 | 1591A/09 | D | KF572836 | 2009 | Beijing, China | complete |
| 70 | 1382A/09 | D | KF572835 | 2009 | Beijing, China | complete |
| 71 | 1357A/09 | D | KF572834 | 2009 | Beijing, China | complete |
| 72 | 2151A/10* | D | KF572839 | 2010 | Beijing, China | complete |
| 73 | 2134A/10 | D | KF572838 | 2010 | Beijing, China | complete |
| 74 | 10574/10 | D | KF572825 | 2010 | Beijing, China | complete |
| 75 | 10290/10 | D | KF572823 | 2010 | Beijing, China | complete |
| 76 | 10285/10 | D | KF572822 | 2010 | Beijing, China | complete |
| 77 | 10108/10 | D | KF572821 | 2010 | Beijing, China | complete |
| 78 | HCoV-OC43/FRA/EPI/Caen/2011/13* | D | KF963242 | 2011 | France | complete |
| 79 | 1783A/10* | E | KF572804 | 2010 | Beijing, China | complete |
| 80 | 2058A/10* | E | KF572809 | 2010 | Beijing, China | complete |
| 81 | 2941A/11* | E | KF572811 | 2011 | Beijing, China | complete |
| 82 | 3074A/12 | E | KF572812 | 2012 | Beijing, China | complete |
| 83 | 3194A/12* | E | KF572814 | 2012 | Beijing, China | complete |
| 84 | CU-H967/2009 | F | JX513258 | 2010 | Thailand | partial |
| 85 | CU-H1367/2010 | F | JX513261 | 2010 | Thailand | partial |
| 86 | CU-H1444/2010 | F | JX513262 | 2010 | Thailand | partial |
| 87 | HCoV-OC43/Niigata.JPN/11-769 | F | AB695085 | 2011 | Japan | partial |
| 88 | HCoV-OC43/Niigata.JPN/11-981 | F | AB695087 | 2011 | Japan | partial |
| 89 | HCoV-OC43/Niigata.JPN/11-768 | F | AB695084 | 2011 | Japan | partial |
| 90 | HCoV-OC43/Niigata.JPN/11-764 | F | AB695083 | 2011 | Japan | partial |
| 91 | HCoV-OC43/Niigata.JPN/11-833 | F | AB695086 | 2011 | Japan | partial |
| 92 | HCoV-OC43/FRA/EPI/Caen/2009/11 | F | KF963240 | 2009 | France | complete |
| 93 | HCoV-OC43/FRA/EPI/Caen/2012/14 | F | KF963243 | 2012 | France | complete |
| 94 | 12691/12 | F | KF572832 | 2012 | Beijing, China | complete |
| 95 | 3269A/12 | F | KF572841 | 2012 | Beijing, China | complete |
| 96 | HCoV-OC43/FRA/EPI/Caen/2013/15 | F | KF963244 | 2013 | France | complete |
| 97 | 12694/12 | G | KF572833 | 2012 | Beijing, China | complete |
| 98 | 12689/12 | G | KF572831 | 2012 | Beijing, China | complete |

* Reference sequences selected to estimate inter-person patristic distances and threshold values.

**References**

1. Vijgen L, Keyaerts E, Moës E *et al*. Complete genomic sequence of human coronavirus OC43: molecular clock analysis suggests a relatively recent zoonotic coronavirus transmission event. *J Virol* 2005; **79**: 1595-1604.

2. St-Jean JR, Jacomy H, Desforges M, Vabret A, Freymuth F, Talbot PJ. Human respiratory coronavirus OC43: genetic stability and neuroinvasion. *J Virol* 2004; **78**: 8824-8834.

3. Vijgen L, Keyaerts E, Lemey P *et al*. Circulation of genetically distinct contemporary human coronavirus OC43 strains. *Virology* 2005; **337**: 85-92.

4. Kin N, Miszczak F, Lin W, Gouilh MA, Vabret A. Genomic analysis of 15 human coronaviruses OC43 (HCoV-OC43s) circulating in France from 2001 to 2013 reveals a high intra-specific diversity with new recombinant genotypes. *Viruses* 2015; **7**: 2358-2377.

5. Zhang Y, Li J, Xiao Y *et al*. Genotype shift in human coronavirus OC43 and emergence of a novel genotype by natural recombination. *J Infect* 2015; **70**: 641-650.

6. Kon M, Watanabe K, Tazawa T *et al.* Detection of human coronavirus NL63 and OC43 in children with acute respiratory infections in Niigata, Japan, between 2010 and 2011. *Jpn J Infect Dis* 2012; **65**: 270-272.

7. Lau SK, Lee P, Tsang AK *et al*. Molecular epidemiology of human coronavirus OC43 reveals evolution of different genotypes over time and recent emergence of a novel genotype due to natural recombination. *J Virol* 2011; **85**: 11325-11337.

8. Suwannakarn K, Chieochansin T, Vichiwattana P, Korkong S, Theamboonlers A, Poovorawan Y. Prevalence and genetic characterization of human coronaviruses in southern Thailand from July 2009 to January 2011. *SE Asian J Trop Med* 2014; **45**: 326-336.
