# Supplementary material for: Identification and evolutionary dynamics of two novel human coronavirus OC43 genotypes associated with acute respiratory infections: phylogenetic, spatiotemporal and transmission network analyses
Source: Emerg Microbes Infect. 2017 Jan 4;6(1):e3–. doi: 10.1038/emi.2016.132 (PMC5285497; doi:10.1038/emi.2016.132)
Supplement: Supplementary Table 4 [file emi2016132x6.docx]

**Supplementary Table S4** Inter-person genetic distances and cutoff values estimated based on a set of HCoV-OC43 S gene reference sequences (*n*=27)

| Genotype | **Genetic Distances*** | | |
| --- | --- | --- | --- |
|  | Lower 2.5% percentile  (95% CI) | Lower 5.0% percentile  (95% CI) | Selected  cutoff value |
| B (n=8) | 0.001 (0.001-0.002) | 0.001 (0.001-0.002) | 0.001 |
| C (n=4) | 0.001 (0.001-0.001) | 0.001 (0.001-0.001) |  |
| D (n=11) | 0.001 (0.001-0.001) | 0.001 (0.001-0.002) |  |
| E (n=4) | 0.001 (0.001-0.001) | 0.001 (0.001-0.001) |  |

* expressed in nucleotide substitutions per site.
